# Supplementary figures and images for: Increased precipitation enhances soil respiration in a semi-arid grassland on the Loess Plateau, China
Source: PeerJ. 2021 Feb 2;9:e10729. doi: 10.7717/peerj.10729 (PMC7863787; doi:10.7717/peerj.10729)

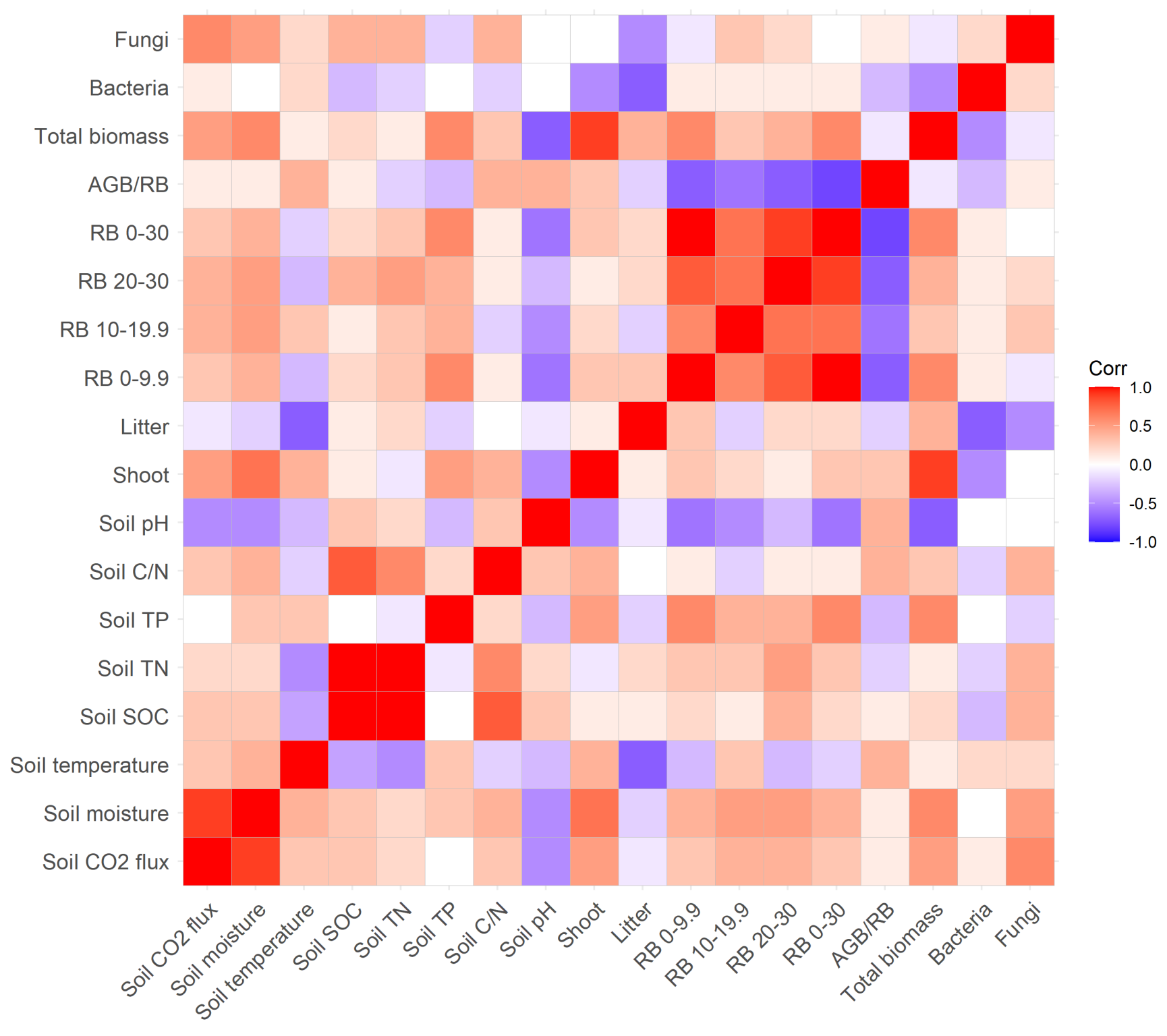

Supplement: Supplemental Information 7 — Red represents positive correlations with blue for negative, and intensity reflects strength of correlation. (Soil C/N: soil organic carbon and total nitrogen ratio). [file peerj-09-10729-s007.pdf]

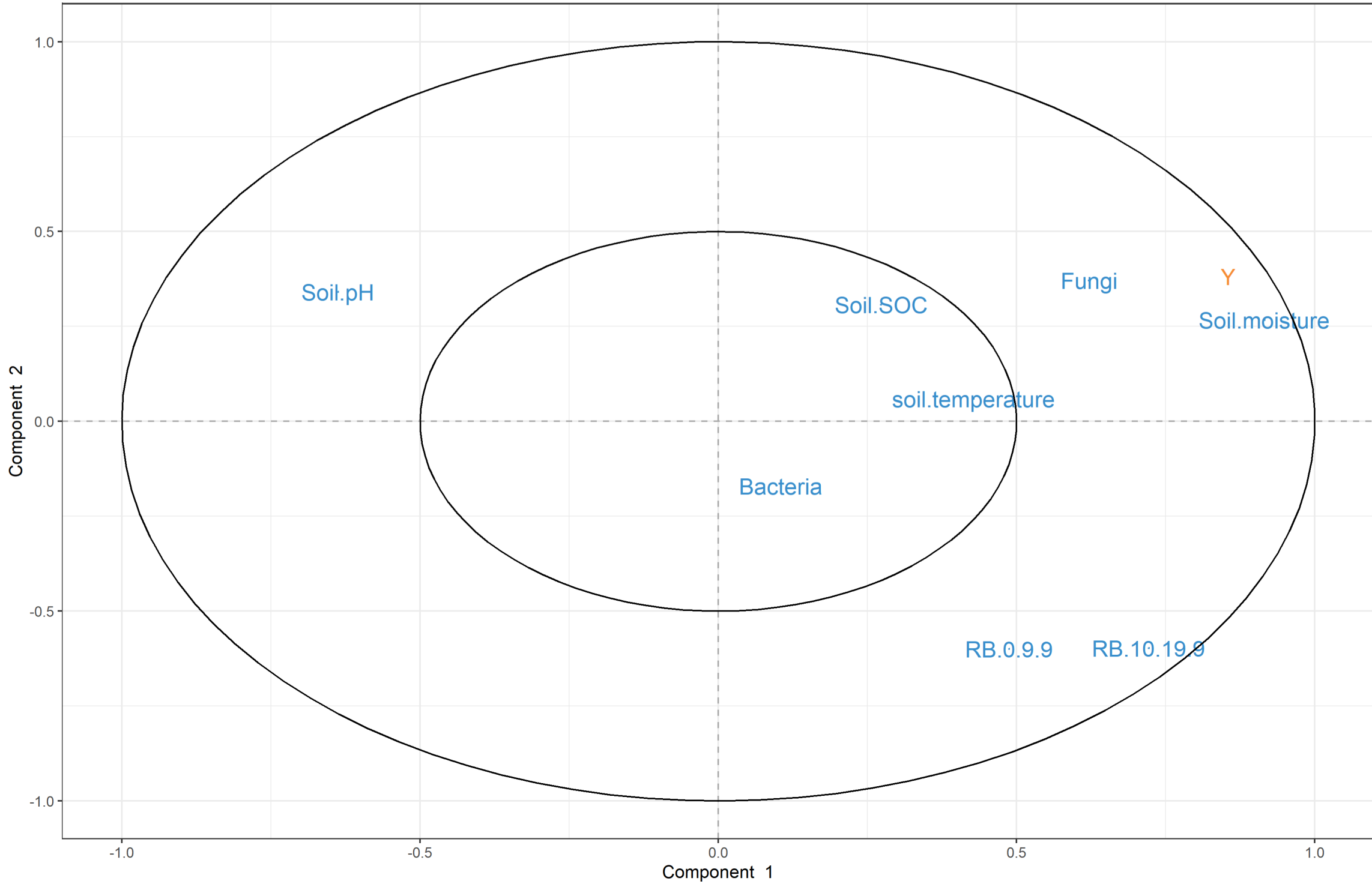

Supplement: Supplemental Information 8 [file peerj-09-10729-s008.pdf]
